# Proteomics- and immunoinformatics-based design of a multi-epitope vaccine ZZ1 against *Glaesserella parasuis*

**DOI:** 10.1186/s12917-026-05621-6

**Published:** 2026-06-12

**Authors:** Zesong Wang, Wenbin Wei, Shifan Feng, Chaoying Jia, Jiyang Fu, Yuanyuan Wang, Yancheng Jin, Hongshuo Liu, Huanchun Chen, Xiangru Wang

**Affiliations:** 1 National Key Laboratory of Agricultural Microbiology, College of Veterinary Medicine, Huazhong Agricultural University, Wuhan, 430070 PR China; 2 Key Laboratory of Preventive Veterinary Medicine in Hubei Province, The Cooperative Innovation Center for Sustainable Pig Production, Wuhan, 430070 PR China; 3 Wuhan Keqian Biological Co., Ltd, Wuhan, 430000 PR China; 4 Engineering Research Center of Animal Biopharmaceuticals, The Ministry of Education of the People’s Republic of China (MOE), Wuhan, 430070 PR China

**Keywords:** *Glaesserella parasuis* (*G. parasuis*), Outer membrane proteins (OMPs), Multi-epitope vaccines (MEVs), Immunoinformatics, Proteomics, Protective efficacy

## Abstract

**Background:**

*Glaesserella parasuis* (*G. parasuis*) is a prevalent opportunistic pathogen of the porcine upper respiratory tract, causing substantial economic losses to the global swine industry. Current commercial vaccines exhibit suboptimal heterologous cross-protection and inherent biosafety concerns, highlighting the need for a safe, broadly effective multi-serotype vaccine.

**Results:**

This study targeted outer membrane proteins (OMPs) with robust cross-serotype immunogenicity from virulent *G. parasuis* strains. Comparative proteomic analysis of five strains identified highly abundant, co-expressed OMPs, which were screened based on antigenicity and physicochemical properties. Epitope prediction for helper T lymphocytes (HTL), cytotoxic T lymphocytes (CTL), and B-cells was performed on selected OMPs. Immunodominant epitopes were concatenated using flexible linkers and fused with the TLR2 agonist phenol-soluble modulin α4 (PSMα4) to engineer a multi-epitope vaccine, designated ZZ1. Screening yielded eight conserved OMPs with high antigenicity, hydrophilicity, and thermostability, from which 6 HTL, 7 CTL, and 11 B-cell epitopes were predicted. Immunoinformatic evaluations revealed that ZZ1 possesses a high antigenicity score of 1.018 (threshold: 0.4) and is non-allergenic and non-toxic. Following successful expression, in vivo trials demonstrated that recombinant ZZ1 elicited robust, specific IgG antibody responses. Challenge tests in piglets revealed 100% protective efficacy against *G. parasuis* serotype 4, and 80% against serotypes 5 and 13.

**Conclusions:**

These findings indicate that ZZ1 is a promising candidate capable of conferring broad cross-protection against multiple prevalent *G. parasuis* serotypes, offering an innovative strategy for next-generation subunit vaccine development.

**Supplementary Information:**

The online version contains supplementary material available at 10.1186/s12917-026-05621-6.

## Background

*G. parasuis* is a commensal opportunistic pathogen colonizing the porcine upper respiratory tract that, under predisposing stress conditions, can breach mucosal barriers to cause *Glässer’s* disease—a systemic infection characterized by fibrinous polyserositis, arthritis, and meningitis, imposing substantial economic losses on global swine production [1–3]. To date, 15 serotypes have been identified, with significant heterogeneity in virulence across and within serotypes [4]. Current epidemiological classifications designate serotypes 1, 5, 10, 12, 13, and 14 as highly virulent variants; serotypes 2, 4, and 15 as moderately pathogenic; and the remaining serotypes as largely avirulent [5].

Vaccination remains the most effective prophylactic strategy against *G. parasuis* infections [6]. However, current commercial multivalent inactivated bacterins exhibit restricted heterologous cross-protection, which is strictly contingent upon antigenic congruence between vaccine strains and circulating field isolates [7]. While these preparations primarily target serotypes 4, 5, and 12, they consistently fail to confer adequate protection against serotype 13—a highly virulent serotype demonstrating increasing prevalence [8–10]. Additionally, traditional bacterins carry inherent biosafety concerns related to incomplete inactivation. These limitations underscore the urgent need for a safe, broadly protective vaccine platform.

OMPs are integral structural constituents of the Gram-negative bacterial envelope that not only mediate essential physiological functions but also exhibit pronounced cross-reactive immunogenicity, establishing them as promising targets for broad-spectrum subunit vaccine development [11, 12]. Several *G. parasuis* OMPs, including PalA, D15, OmpP2, and GAPDH, have demonstrated robust immunogenicity [11, 13]. Nevertheless, recombinant subunit vaccines based on individual proteins often suffer from suboptimal immunogenicity and restricted cross-serotype protection [7]. To overcome these limitations, immunoinformatics-driven multi-epitope vaccine (MEV) design has emerged as a transformative strategy, enabling the rational identification and combinatorial assembly of immunodominant B-cell and T-cell epitopes to elicit focused, synergistic humoral and cellular immune responses [14, 15]. This reverse vaccinology approach has been successfully applied against diverse bacterial and viral pathogens, including *Brucella spp*., *Mycoplasma pneumoniae*, and *Salmonella Typh*i, yet remains largely unexplored for *G. parasuis* [16–18].

In this study, we combined comparative proteomic analysis of OMPs from five virulent *G. parasuis* strains representing serotypes 4, 5, and 13 with an immunoinformatics pipeline to design a MEV candidate, designated ZZ1. Following in silico structural validation, the vaccine was expressed in Escherichia coli and evaluated for immunogenicity and cross-protective efficacy in a porcine challenge model.

## Materials and methods

### Bacterial strains, culture media and chemicals

*Escherichia coli* (*E. coli*) BL21(DE3) strain was utilized as host cells for recombinant protein expression. The *E. coli* strain was cultured in Luria-Bertani (LB) medium supplemented with 100 µg/mL ampicillin. The *G. parasuis* isolates utilized in this study (strains HB04, SJZ05, and GD20) are characterized by high virulence and robust immunogenicity. Representing serotypes 4, 5, and 13, respectively, these clinical strains were isolated from distinct commercial swine herds in the Chinese provinces of Hubei, Hebei, and Guangdong. Additionally, *G. parasuis* strains MD0322 (serotypes 4) and SH0165 (serotypes 5) were obtained from our laboratory’s standard collection. All *G. parasuis* strains were cultured at 37 °C on tryptic soy agar (TSA) or in tryptic soy broth (TSB) (Difco Laboratories, Detroit, MI, USA), intrinsically supplemented with 10 µg/mL nicotinamide adenine dinucleotide (NAD) and 5% bovine serum. OMPs were isolated using a commercial bacterial OMP extraction kit (Bjbalb, Beijing, China).

### OMP extraction, proteomic analysis, and target protein selection

OMPs were successfully extracted from the five *G. parasuis* isolates (HB04, SJZ05, GD20, MD0322, and SH0165) and submitted to APTBIO (Shanghai, China) for 4D label-free quantitative proteomic analysis. OMPs conserved across all five strains were prioritized as the core dataset for downstream bioinformatic evaluation.

To assess their suitability as vaccine candidates, the ExPASy ProtParam tool (http://web.expasy.org/protparam/, accessed on 24 October 2023) was employed to compute key physicochemical properties, including sequence length, molecular weight, theoretical isoelectric point (pI), and the grand average of hydropathicity (GRAVY) index. Concurrently, the VaxiJen v2.0 server (http://www.ddg-pharmfac.net/vaxijen/VaxiJen/VaxiJen.html, accessed on 19 October 2023) was used to quantify predicted antigenicity [19]. Ultimately, eight core OMPs demonstrating superior antigenicity, favorable hydrophilicity, and robust structural stability were selected as the primary targets for MEV design.

### T-cell and B-cell epitope prediction and filtering

A systematic immunoinformatic pipeline was utilized to predict linear B-cell, cytotoxic T-lymphocyte (CTL), and helper T-lymphocyte (HTL) epitopes from the eight selected OMPs.

#### B-cell epitopes

Linear B-cell epitopes, which are crucial for eliciting a neutralizing humoral response, were predicted using the BepiPred-2.0 algorithm via the IEDB server (http://tools.iedb.org/main/bcell/, accessed on 25 November 2023) [20].

#### CTL epitopes

To accurately reflect the porcine immune system, Swine Leukocyte Antigen (SLA) alleles (specifically SLA-1*0401) were utilized to predict 9-mer CTL epitopes via the IEDB server (http://tools.iedb.org/mhci/, accessed on 23 November 2023). To ensure robust prediction, these results were cross-referenced with the NetCTL-1.2 server (https://services.healthtech.dtu.dk/services/NetCTL-1.2/, accessed on 23 November 2023) using default settings (threshold: 0.75) [20, 21].

#### HTL epitopes

Similarly, 15-mer HTL epitopes were predicted using both the IEDB server (http://tools.iedb.org/mhcii/, accessed on 24 November 2023) and the NetMHCII-2.3 server (https://services.healthtech.dtu.dk/services/NetMHCII-2.3/, accessed on 24 November 2023) [22, 23].

For all T-cell and B-cell predictions, overlapping epitopes predicted by multiple servers were prioritized. Subsequently, these candidate epitopes were rigorously filtered based on antigenicity (VaxiJen v2.0), allergenicity (AllergenFP: https://ddg-pharmfac.net/AllergenFP), and toxicity (ToxinPred: https://webs.iiitd.edu.in/raghava/toxinpred/design.php) [24]. Only epitopes confirmed to be highly antigenic, strictly non-allergenic, and non-toxic were selected for final vaccine assembly.

### MEV construction and physicochemical profiling

The novel MEV, designated ZZ1, was engineered by rationally concatenating the selected epitopes. To ensure optimal spatial configuration and independent folding, specific short amino acid linkers were utilized: GPGPG for HTL epitopes, AAY for CTL epitopes, and KK for B-cell epitopes [25]. To further augment innate immune recognition, the Toll-like receptor 2 (TLR2) agonist PSMα4 (Accession no. A9JX08) was incorporated at the N-terminus as an intramolecular adjuvant [26]. This adjuvant was fused to the first HTL epitope via a rigid EAAAK linker to prevent steric hindrance [27]. Following assembly, the full-length ZZ1 chimera was re-evaluated using VaxiJen v2.0, ToxinPred, AllergenFP v1.0, and ProtParam to confirm that the global construct retained excellent antigenicity, safety (non-toxic/non-allergenic), and requisite physicochemical stability.

### Structural modeling, validation, and conformational epitope prediction

The secondary structural elements of the ZZ1 construct were predicted utilizing the SOPMA server (https://npsa-prabi.ibcp.fr/cgi-bin/npsa_automat.pl?page=npsa%20_sopma.html, accessed on 14 March 2024), which employs multiple sequence alignments and neural network algorithms to calculate the spatial probability of specific residues [28]. Initial de novo tertiary structure modeling was performed using the Robetta server (https://robetta.bakerlab.org/, accessed on 19 March 2024) [29], and the selected model was subsequently subjected to dynamic refinement via the GalaxyRefine server (https://galaxy.seoklab.org/cgi-bin/submit.cgi?type=REFINE, accessed on 20 March 2024) [30]. The stereochemical quality of the refined 3D model was validated using the SWISS-MODEL Structure Assessment tool (https://swissmodel.expasy.org/assess, accessed on 21 March 2024) and ProSA-web (https://prosa.services.came.sbg.ac.at/prosa.php, accessed on 21 March 2024), with reliability assessed via Z-scores and Ramachandran plot residue distributions [31, 32]. Finally, structural (conformational) B-cell epitopes mapped to the refined 3D model were predicted utilizing the ElliPro server (http://tools.iedb.org/ellipro/, accessed on 27 March 2024) based on residue prominence indices (PIs) [33].

### Recombinant plasmid construction and in vitro expression

The amino acid sequence of the ZZ1 vaccine was reverse-translated, and the resultant nucleotide sequence was optimized for *E. coli* codon bias by GenScript (Nanjing, China). The optimized gene, flanked by NdeI and HindIII restriction sites at the 5′ and 3′ ends, respectively, was theoretically assembled into the pET-22b(+) expression vector using SnapGene software (v. 6.0; GSL Biotech) before physical synthesis and cloning.

The resulting pET-22b(+)-ZZ1 recombinant plasmid was transformed into *E. coli* BL21(DE3) chemically competent cells. Positive transformants were initially inoculated into 4 mL of LB medium containing 100 µg/mL ampicillin and incubated overnight at 37 °C with shaking (180 rpm). This seed culture was scaled up by transferring it to 400 mL of fresh LB medium (1:100 ratio) and grown at 37 °C until the optical density at 600 nm (OD_600_) reached 0.5–0.6. Protein expression was then induced via the addition of 0.5 mM isopropyl β-D-1-thiogalactopyranoside (IPTG) for 5 h. Harvested cell pellets were resuspended and subjected to three cycles of lysis using a high-pressure cell disruptor. Following centrifugation (9000 rpm, 25 min, 4 °C), the supernatant was collected, and the soluble ZZ1 recombinant protein was purified utilizing nickel-nitrilotriacetic acid (Ni-NTA) affinity chromatography targeting the C-terminal 6 × His tag. Protein molecular weight and purity were ultimately verified via sodium dodecyl sulfate-polyacrylamide gel electrophoresis (SDS-PAGE).

### Vaccination and challenge of pigs

Thirty healthy, crossbred weaned piglets (aged 21–26 days) were purchased from a commercial farm in Hubei Province, China, which is a *G. parasuis*‑free facility. The piglets were housed with ad libitum feed and water. No formal sample size calculation was performed; group size (*n* = 5/group, total 30) was based on similar studies. Piglets were of mixed sex, weighing 5.5–7.0 kg. Animals showing illness before immunization were excluded and replaced; none were excluded after randomization.

The piglets were randomly assigned to six groups (A–F; *n* = 5 per group) using a computer-generated random number sequence (Microsoft Excel, RAND function). The test groups (Groups A–C) were immunized intramuscularly with 2 mL of the vaccine formulation, comprising 200 µg of the purified ZZ1 protein thoroughly emulsified in Montanide™ ISA 201 VG adjuvant, a water-in-oil-in-water (w/o/w) emulsion that combines the depot effect of oily adjuvants with improved injectability and safety, and has been widely used in swine vaccines to induce strong and durable humoral as well as cell-mediated immune responses. The control groups (Groups D–F) received an equivalent volume of sterile phosphate-buffered saline (PBS) emulsified with the same adjuvant. The experimenter who performed the immunizations, clinical scoring, sample collection, and data analysis was blinded to the group allocation. The vaccine and control preparations were visually identical. A homologous booster dose was administered 21 days post-primary immunization. Fourteen days following the booster (Day 35), piglets were challenged via intrapleural injection with highly virulent *G. parasuis* strains at the following predefined doses: HB04 (Group A & D: 1.1 × 10^10^ colony-forming units (CFU)/pig), SJZ05 (Group B & E: 5.0 × 10^9^ CFU/pig), and GD20 (Group C & F: 1.3 × 10^10^ CFU/pig).

Post-challenge, animals were continuously monitored for 7 days. Clinical symptoms, behavioral changes, and mortalities were systematically recorded. To adhere to ethical guidelines, animals exhibiting moribund conditions (e.g., severe dyspnea combined with an inability to rise) were humanely euthanized and recorded as mortalities for that day. Surviving animals were euthanized at the conclusion of the observation period. Euthanasia was performed by intravenous overdose of sodium pentobarbital at a dose of approximately 120 mg/kg body weight, inducing rapid unconsciousness and cardiac/respiratory arrest, consistent with AVMA guidelines and institutional approval. The protective efficacy of the ZZ1 vaccine was evaluated comprehensively based on survival rates, reduction of clinical symptoms, and gross pathological/histopathological findings at necropsy.

### Serum antibody detection

To evaluate the humoral immune response, whole blood was collected from the anterior vena cava prior to primary immunization (Day 0), prior to the booster (Day 21), and prior to the challenge (Day 35). Sera were separated via centrifugation and stored at -80 °C. ZZ1-specific serum IgG concentrations were quantified using a standardized indirect enzyme-linked immunosorbent assay (ELISA) [34]. Briefly, 96-well microtiter plates were coated with purified recombinant ZZ1 protein at 4 µg/mL (100 µL/well) and incubated overnight at 4 °C. Plates were blocked with 5% bovine serum albumin (BSA) at 37 °C for 2 h, washed five times with PBS containing 0.05% Tween-20 (PBST), and stored at 4 °C until use. All serum samples were diluted 1:200 in PBS, added to the wells (100 µL/well), and incubated for 30 min at 37 °C. Following five PBST washes, a horseradish peroxidase (HRP)-conjugated rabbit anti-porcine IgG secondary antibody (1:4000 dilution; Biodragon, Beijing, China) was added and incubated for 30 min at 37 °C. The reaction was developed in the dark for 10 min utilizing a 3,3′,5,5′-Tetramethylbenzidine (TMB) substrate solution (Keqian Biological Co., Wuhan, China). Absorbance was subsequently recorded at 450 nm using a Tecan M200 microplate reader.

### Statistical analysis

All statistical analyses and graphical representations were performed using GraphPad Prism software (version 8.0). Data are expressed as the mean ± standard deviation (SD). Differences in antibody titers between the vaccinated and control groups across multiple time points were analyzed using a two-way repeated-measures analysis of variance (ANOVA). Upon detection of a significant interaction between time and treatment group, Sidak’s multiple comparisons post hoc test was applied to evaluate statistical significance at specific time points. Statistical significance in the figures is denoted as follows: * *p* < 0.05, ** *p* < 0.01, and *** *p* < 0.001.

## Results

### Proteomic analysis of OMPs

**Fig. 1:**
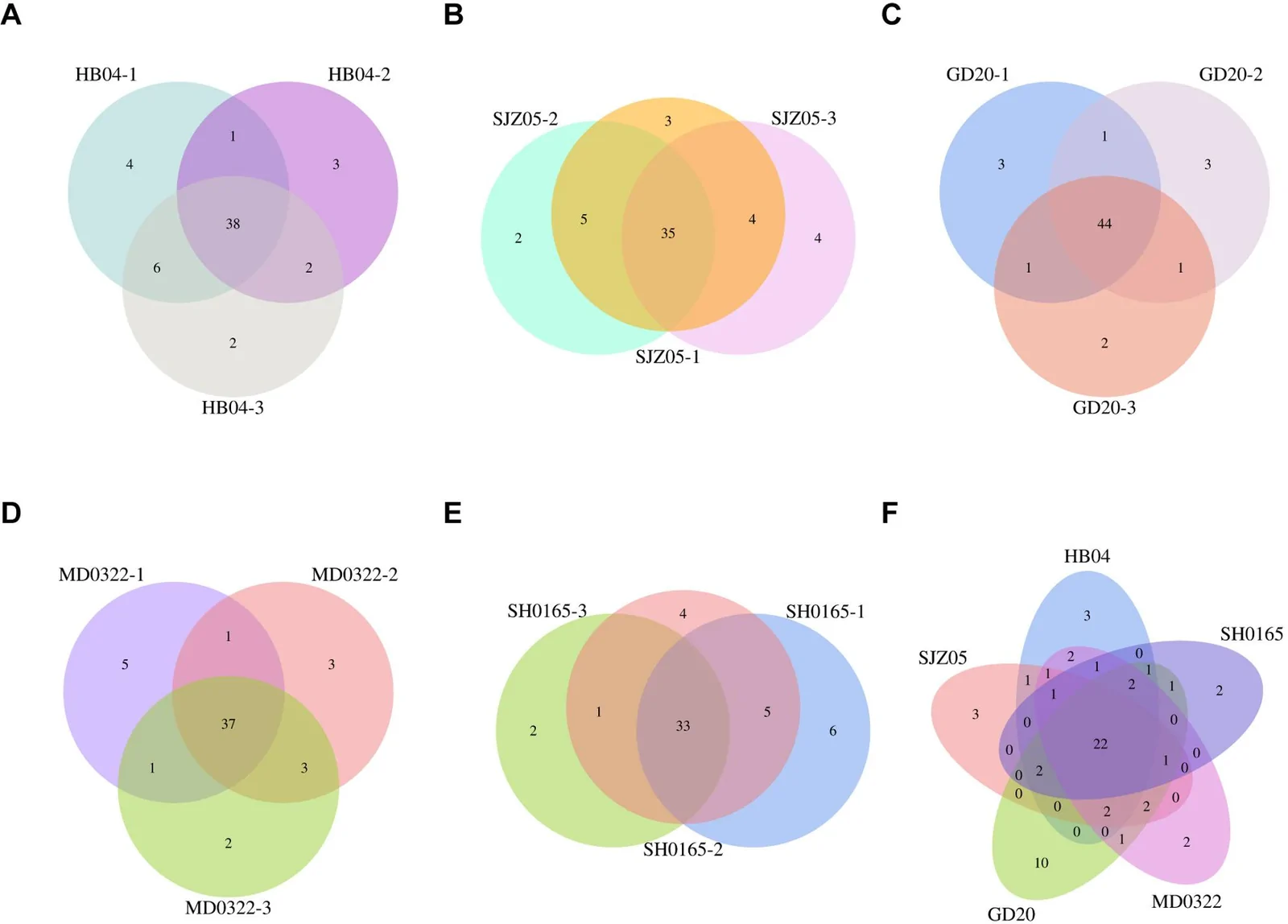
Proteomic identification of OMPs across five clinical *G. parasuis* strains. **A**–**E** Numbers of OMPs identified in strains HB04 (38), SJZ05 (35), GD20 (44), MD0322 (37), and SH0165 (33). **F** Venn diagram showing a core set of 22 OMPs conserved across all five isolates

Following 4D label-free quantitative proteomic analysis, the clinical *G. parasuis* isolates (strains HB04, SJZ05, GD20, MD0322 and SH0165) were found to express 38, 35, 44, 37, and 33 OMPs, respectively (Fig. 1A-E; see Additional file 1 for detailed data). Comparative analysis revealed a core set of 22 OMPs that were comprehensively conserved across all five strains (Fig. 1F).

### Target proteins identification

**Fig. 2:**
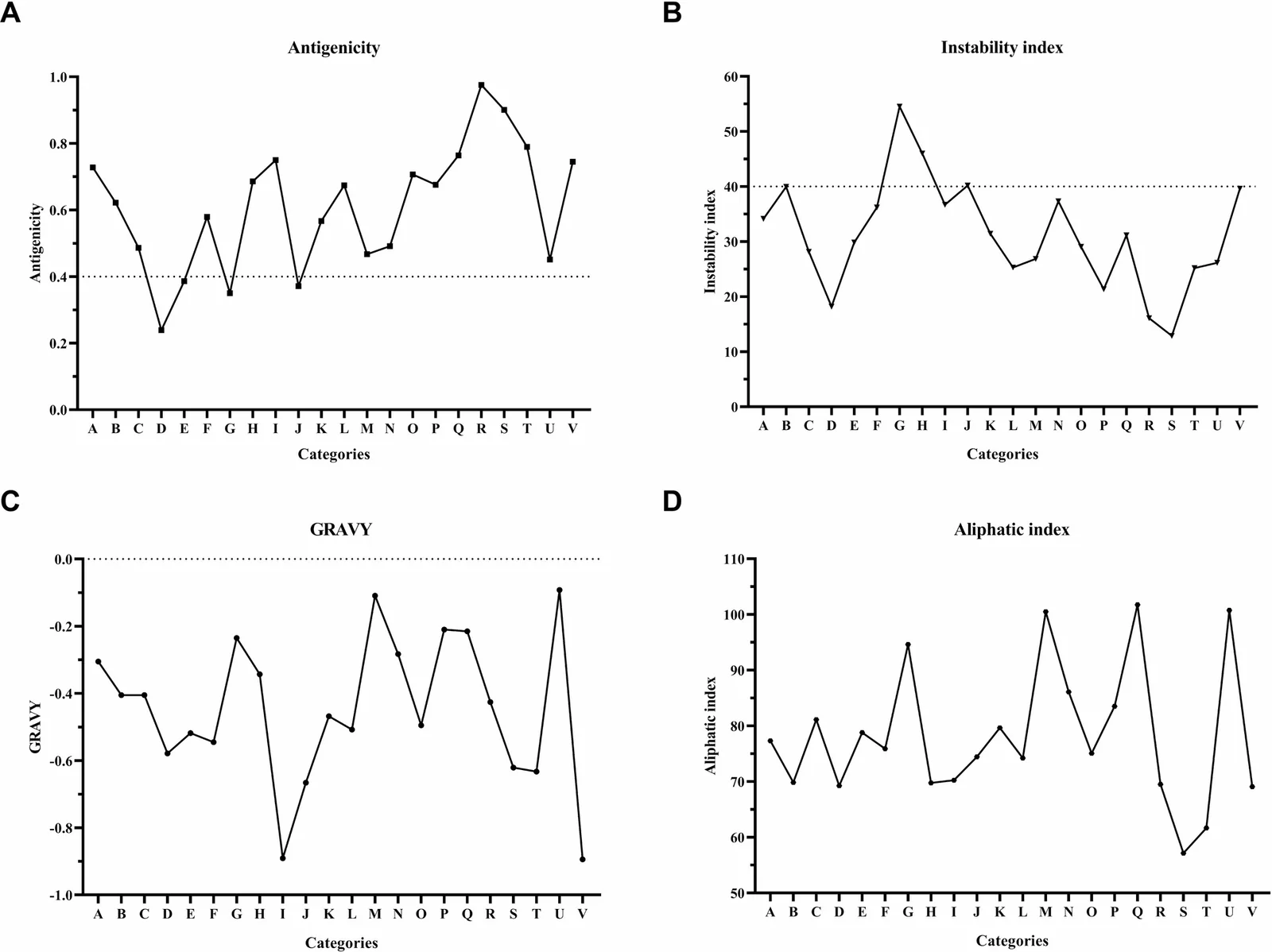
In silico prioritization of candidate target proteins through integrated antigenicity and physicochemical profiling. **A** Antigenicity scores of the 22 conserved OMPs (VaxiJen v2.0, threshold > 0.4). **B** Instability index (values < 40 indicate stability). **C** Aliphatic index (thermal stability). **D** GRAVY index (negative values denote hydrophilicity). Analyses **B**–**D** were performed using ExPASy ProtParam

The 22 conserved OMPs were subjected to bioinformatic analysis to evaluate their antigenicity (Fig. 2A), instability index (Fig. 2B), aliphatic index (Fig. 2C), and grand average of hydropathy (GRAVY; Fig. 2D) [see Additional file 2 (Table S1)]. Based on a comprehensive assessment of these parameters, eight OMPs demonstrating high antigenicity, favorable hydrophilicity, and robust structural stability were selected as optimal candidate targets. The antigenicity scores and key physicochemical properties of these eight prioritized proteins are summarized in Table 1.

**Table 1:** Antigenicity and key physicochemical properties of target proteins

Uniprot ID	Antigenicity	Theoretical pI	Instability index	Aliphatic index	GRAVY
A0A806JA38	0.7279	6.28	34.12	77.32	-0.305
A0A859IFM7	0.7496	5.45	36.70	70.25	-0.891
B8F6K9	0.6739	6.28	25.29	74.23	-0.508
B8F4Z5	0.7066	5.04	29.05	75.07	-0.495
D9IR01	0.6758	9.26	21.34	83.51	-0.210
F2GDE3	0.9757	9.04	16.10	69.50	-0.426
A0A0M3U1B8	0.7893	6.31	25.18	61.65	-0.633
A0A8A2WC79	0.7448	8.51	39.62	69.09	-0.894

### Linear epitope prediction

The eight candidate OMPs were systematically screened to identify helper T lymphocyte (HTL), cytotoxic T lymphocyte (CTL), and linear B-cell epitopes. Epitopes demonstrating high antigenicity, strict non-allergenicity, and non-toxicity were prioritized for downstream assembly. Through this rigorous filtering process, a total of 6 HTL, 7 CTL, and 11 B-cell epitopes were successfully predicted (Tables 2, 3 and 4).

**Table 2:** The immunodominant HTL epitopes of the target proteins

Uniprot ID	Position	HTL epitopes	Antigenicity
A0A859IFM7	395–409	AGLYTRANGHKSSHG	1.2652
B8F4Z5	758–772	INSLVGSIGAKAQYQ	0.8003
D9IR01	166–180	GAGVEYAILPELAFR	1.6785
A0A0M3U1B8	273–287	EGGVYGPKAEEMAGK	1.1271
	227–241	SDKEKTIYTVNADIR	0.9917
A0A8A2WC79	118–132	GKEIKLAPVNGSNSN	1.6779

**Table 3:** The immunodominant CTL epitopes of the target proteins

Uniprot ID	Position	CTL epitopes	Antigenicity
A0A806JA38	43–51	SVQDLQTRY	1.2898
	120–128	ASNVATVSY	0.7831
A0A859IFM7	437–445	AVSASNQHY	0.5060
B8F6K9	534–542	SSDSYKHKF	1.4416
	624–632	KLDFKSQQY	1.5367
A0A0M3U1B8	60–68	ELMEPALGY	0.5589
	492–500	ALDSGSSRY	1.2049

**Table 4:** The immunodominant B-cell epitopes of the target proteins

Uniprot ID	Position	B-cell epitopes	Antigenicity
A0A806JA38	54–69	VYFDFDSYTLKAEDQQ	1.4061
A0A859IFM7	461–480	GNNPHRDSVNGKGWKQLSDL	0.9933
B8F6K9	621–635	KEMKLDFKSQQYHTH	1.2006
B8F4Z5	336–355	MLNNIHTNGNNSNITIVRDN	0.9104
	571–590	LNSIRNRLEHSRSSNAYPYL	0.7949
D9IR01	45–64	QIDSKYANDARYDATNLKYG	1.1516
	191–210	NFTKAEAKENRRATYNYSPD	1.0965
F2GDE3	211–230	LDRTDYHANNANNGSSRRTT	1.3530
A0A0M3U1B8	172–195	NEEGRPNYLHDDYYTNVKGKRAGV	1.1324
	424–442	SKENNWVATADDDRKAGY	0.9246
A0A8A2WC79	28–48	GHQQAVDKTENPITNQQVSEN	0.7953

### ZZ1 vaccine construct design

**Fig. 3:**
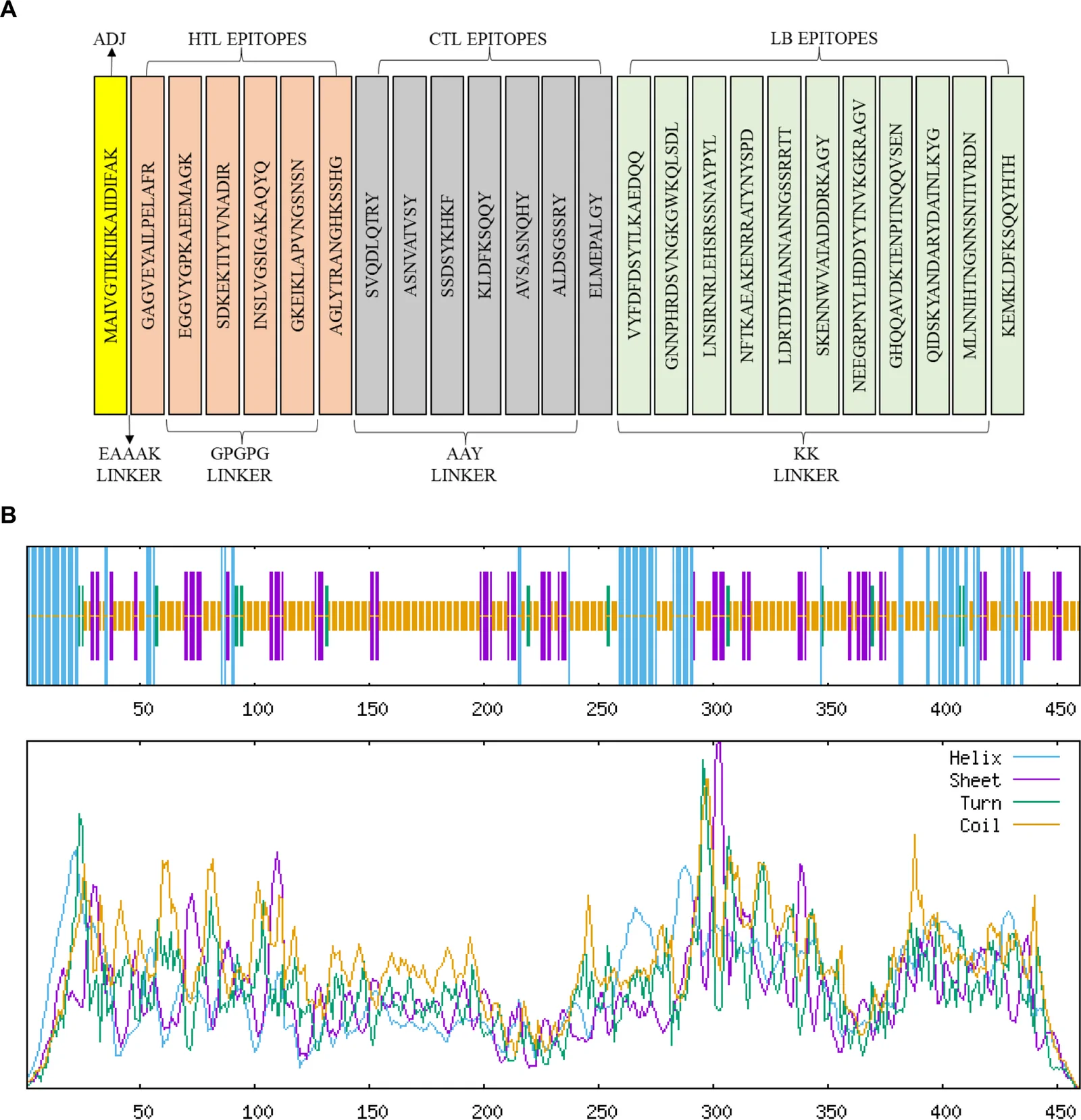
Schematic representation of the MEV ZZ1 construct and secondary structure. **A** Linear architecture of the ZZ1 chimera: N-terminal adjuvant PSMα4 (yellow), HTL epitopes (orange-pink), CTL epitopes (gray), and B‑cell epitopes (light green). **B** Secondary structure prediction across residues 1–460: α-helices (blue), extended strands (purple), β-turns (green), and random coils (yellow)

The MEV construct, designated ZZ1, was engineered by integrating the selected 6 HTL, 7 CTL, and 11 B-cell epitopes. To ensure optimal structural flexibility and appropriate epitope spatial presentation, GPGPG, AAY, and KK linkers were utilized to covalently connect the HTL, CTL, and B-cell epitopes, respectively. To further enhance intrinsic immunogenicity, the TLR2 agonist PSMα4 was incorporated as an intramolecular adjuvant at the N-terminus, joined to the first HTL epitope via a rigid EAAAK linker (Fig. 3A). The finalized ZZ1 vaccine sequence comprised 460 amino acids.

### Immunoinformatic analysis of the ZZ1 vaccine

The overall antigenicity of the ZZ1 construct was evaluated using the VaxiJen v2.0 server, which predicted a high score of 1.018 in the bacterial model, significantly exceeding the standard threshold of 0.4. Concurrent allergenicity and toxicity analyses, conducted via the AllergenFP v1.0 and ToxinPred servers, confirmed that the vaccine is both non-allergenic and non-toxic. Physicochemical profiling using the ProtParam server estimated the in vivo half-life of ZZ1 to be 30 h in mammalian reticulocytes, > 20 h in yeast, and > 10 h in *E. coli*. Furthermore, the computed instability index was 28.14 (well below the instability threshold of 40), classifying the protein as structurally stable. The aliphatic index and GRAVY values were recorded at 57.41 and − 0.973, respectively, confirming that the ZZ1 vaccine possesses excellent thermostability and hydrophilicity.

### ZZ1 secondary structure prediction

Secondary structure prediction for the ZZ1 vaccine was performed using the SOPMA server. The analysis revealed that the construct comprises 19.78% alpha helices, 4.57% beta turns, 56.96% random coils, and 18.70% extended strands (Fig. 3B). The pronounced predominance of random coil conformations is highly favorable for structural flexibility, which facilitates the optimal surface exposure of antigenic epitopes for subsequent immune recognition.

### ZZ1 tertiary structure modeling and validation

**Fig. 4:**
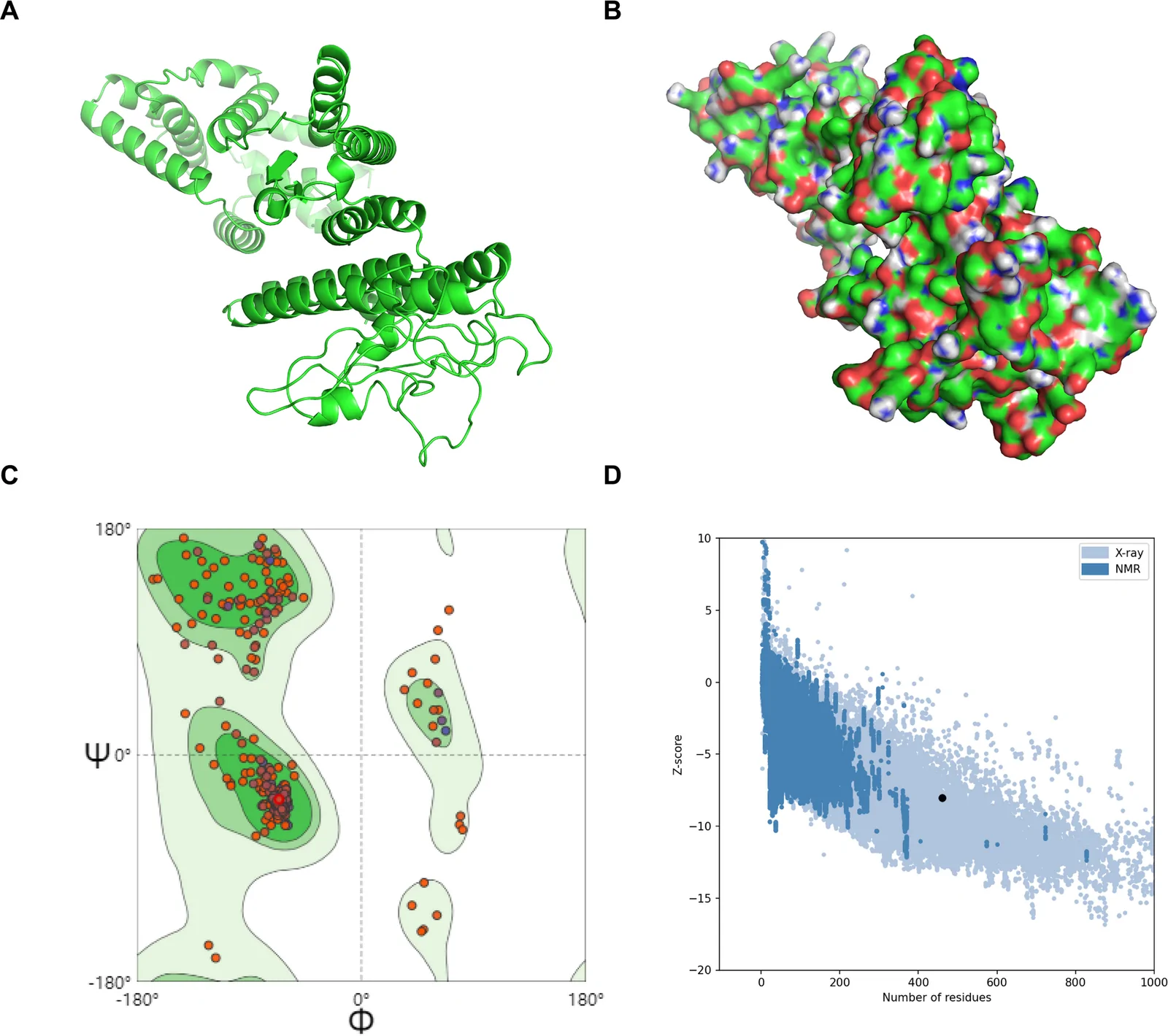
Tertiary structure modeling and stereochemical validation of the ZZ1 vaccine. **A–B** Refined 3D model in cartoon (**A**) and surface (**B**) representation. **C** Ramachandran plot: 94.10% of residues in favored regions, 0.27% in outliers. **D** ProSA Z‑score of −8.02, within the range for native proteins of comparable size

Initial tertiary structure modeling of the ZZ1 vaccine was generated using the Robetta server, followed by dynamic structural optimization via the GalaxyRefine server. The refined 3D model was subsequently visualized using PyMOL (Fig. 4A–B). Comprehensive stereochemical evaluation of the refined model was conducted utilizing the SWISS-MODEL Structure Assessment tool and ProSA-web. The resulting Ramachandran plot demonstrated that 94.10% of the amino acid residues were located in stereochemically favored regions, with merely 0.27% present in outlier regions (Fig. 4C). Additionally, the calculated Z-score of -8.02 fell well within the acceptable range for native proteins of comparable size (Fig. 4D). Together, these validation metrics verify that the optimized ZZ1 3D model possesses high structural quality and reliability.

### Conformational B-cell epitope prediction

**Fig. 5:**
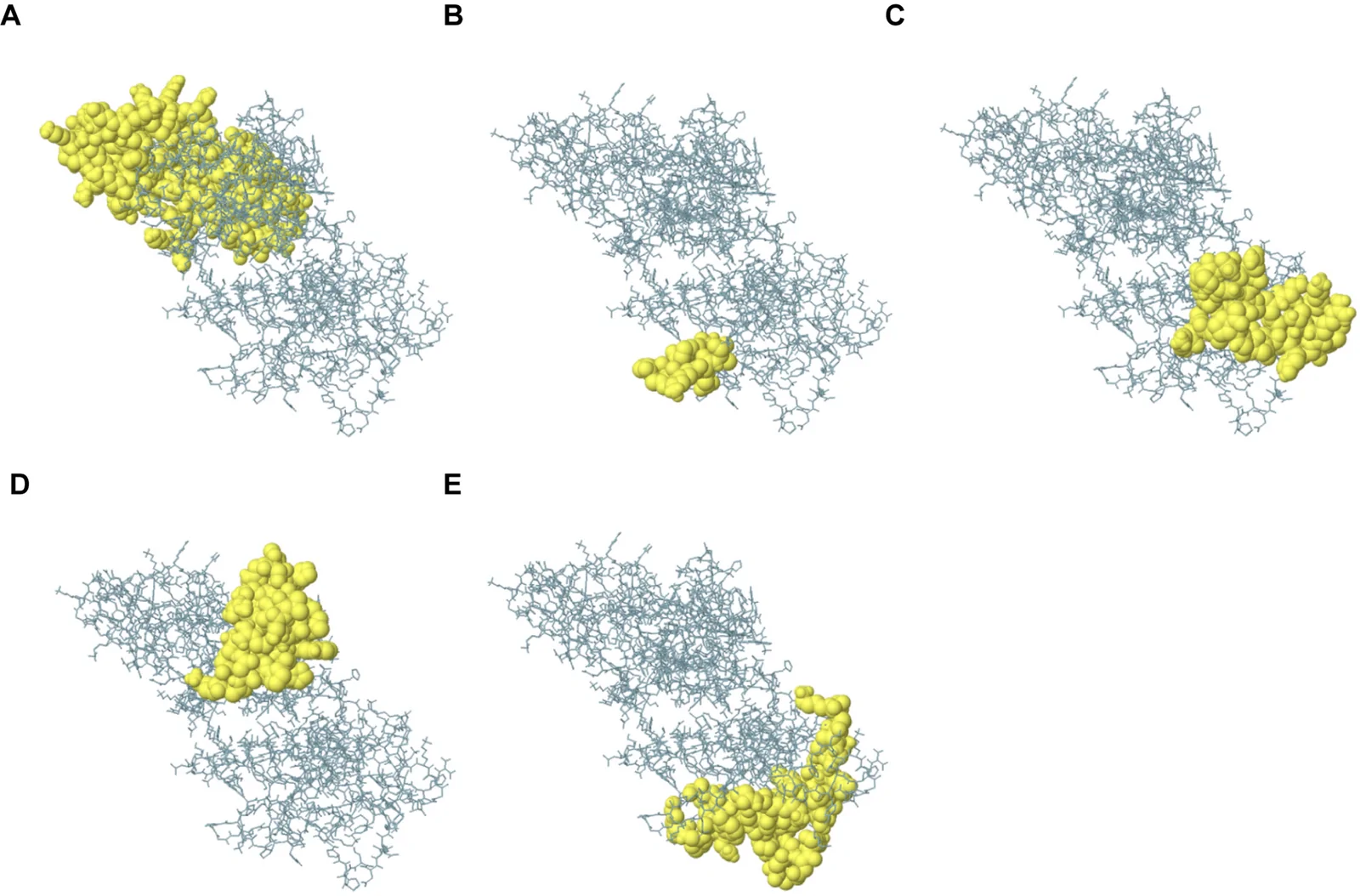
Conformational B-cell epitope mapping on the 3D structure of the ZZ1 vaccine. **A**–**E** Five discontinuous epitopes predicted by ElliPro (yellow regions); the protein backbone is shown in gray

Discontinuous (conformational) B-cell epitope prediction revealed that the ZZ1 construct harbors five conformational epitopes. These structural epitopes range in length from 10 to 106 amino acid residues, with prediction scores spanning from 0.599 to 0.781 (Table 5; Fig. 5A–E).

**Table 5:** The B-cell conformational epitopes of ZZ1 vaccine

No.	Residues	Number of residues	Score
A	A: N323, A: G324, A: S325, A: S326, A: R327, A: R328, A: T329, A: T330, A: K331, A: K332, A: S333, A: K334, A: E335, A: N336, A: N337, A: W338, A: V339, A: T341, A: A342, A: D345, A: R346, A: A348, A: G349, A: Y350, A: K351, A: K352, A: N353, A: E354, A: E355, A: G356, A: R357, A: P358, A: N359, A: Y360, A: L361, A: H362, A: D363, A: D364, A: Y365, A: Y366, A: T367, A: N368, A: V369, A: K370, A: G371, A: K372, A: R373, A: A374, A: G375, A: V376, A: K377, A: K378, A: G379, A: H380, A: Q381, A: Q382, A: S405, A: K406, A: Y407, A: D410, A: A411, A: R412, A: D414, A: A415, A: L418, A: K419, A: G421, A: K422, A: K423, A: N424, A: F425, A: T426, A: K427, A: A428, A: E429, A: A430, A: K431, A: E432, A: N433, A: R434, A: R435, A: A436, A: T437, A: Y438, A: N439, A: Y440, A: S441, A: P442, A: D443, A: K444, A: K445, A: K446, A: E447, A: M448, A: K449, A: L450, A: D451, A: F452, A: K453, A: S454, A: Q455, A: Q456, A: Y457, A: H458, A: T459, A: H460	106	0.781
B	A: Q100, A: G101, A: P102, A: G103, A: P104, A: G105, A: G106, A: K107, A: E108, A: I109	10	0.765
C	A: K110, A: L111, A: A112, A: P113, A: V114, A: N115, A: G116, A: S117, A: N118, A: S119, A: N120, A: G121, A: P122, A: G123, A: P124, A: G125, A: A126, A: G127, A: L128, A: Y129, A: T130, A: R131, A: A132, A: N133, A: G134, A: H135, A: K136, A: S137, A: S138, A: H139	30	0.723
D	A: G223, A: Y224, A: K225, A: D240, A: K243, A: K244, A: G245, A: N246, A: N247, A: P248, A: H249, A: R250, A: D251, A: S252, A: V253, A: N254, A: G255, A: K256, A: G257, A: W258, A: K259, A: Q260, A: L261, A: L264, A: K287, A: N291, A: N292, A: I293, A: H294, A: T295, A: N296, A: G297, A: N298, A: N299, A: S300, A: N301, A: I302	37	0.684
E	A: A23, A: A24, A: K25, A: G26, A: A27, A: G28, A: V29, A: E30, A: Y31, A: G41, A: P42, A: G43, A: P44, A: G45, A: E46, A: G47, A: G48, A: V49, A: Y50, A: G51, A: P52, A: K53, A: A54, A: E55, A: E56, A: M57, A: A58, A: G59, A: K60, A: G61, A: P62, A: G63, A: P64, A: G65, A: S66, A: D67, A: K68, A: K70, A: T71, A: I72, A: I79, A: G81, A: P82, A: G83, A: P84, A: I86, A: N87, A: S88, A: L89, A: V90, A: G91, A: S92, A: I93, A: G94, A: A95, A: K96, A: A97, A: Q98, A: Y99	59	0.599

### Recombinant plasmid construction and in vitro expression

**Fig. 6:**
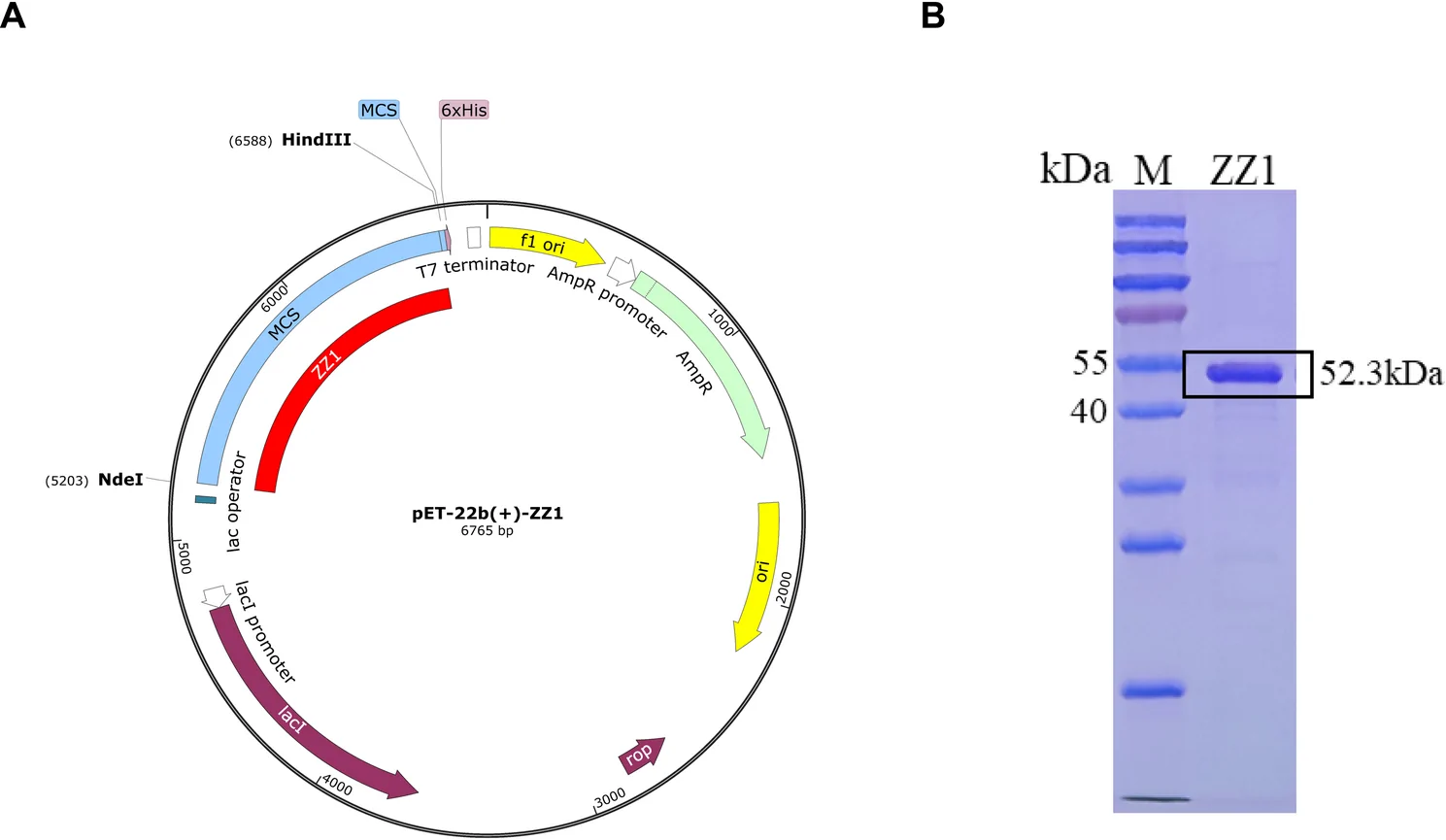
Construction of the recombinant plasmid and in vitro expression of the ZZ1 vaccine. **A** In silico cloning map of the codon‑optimized ZZ1 gene inserted into pET‑22b(+) via NdeI and HindIII. **B** SDS‑PAGE confirming expression and purification of ZZ1 (expected size: 52.3 kDa) in *E. coli* BL21(DE3)

The amino acid sequence of the ZZ1 vaccine was reverse-translated and subjected to codon optimization by GenScript for enhanced heterologous expression in *E. coli*. The optimized nucleotide sequence was engineered to include NdeI and HindIII restriction enzyme cleavage sites at the 5′ and 3′ ends, respectively. Following in silico sequence assembly and validation using SnapGene software, the synthesized gene was physically cloned into the pET-22b(+) expression vector (Fig. 6A). The recombinant ZZ1 protein, exhibiting the expected molecular weight of 52.3 kDa, was successfully expressed in E. coli and its subsequent purification was verified via SDS-PAGE (Fig. 6B).

### Antibody response in vaccinated piglets

**Fig. 7:**
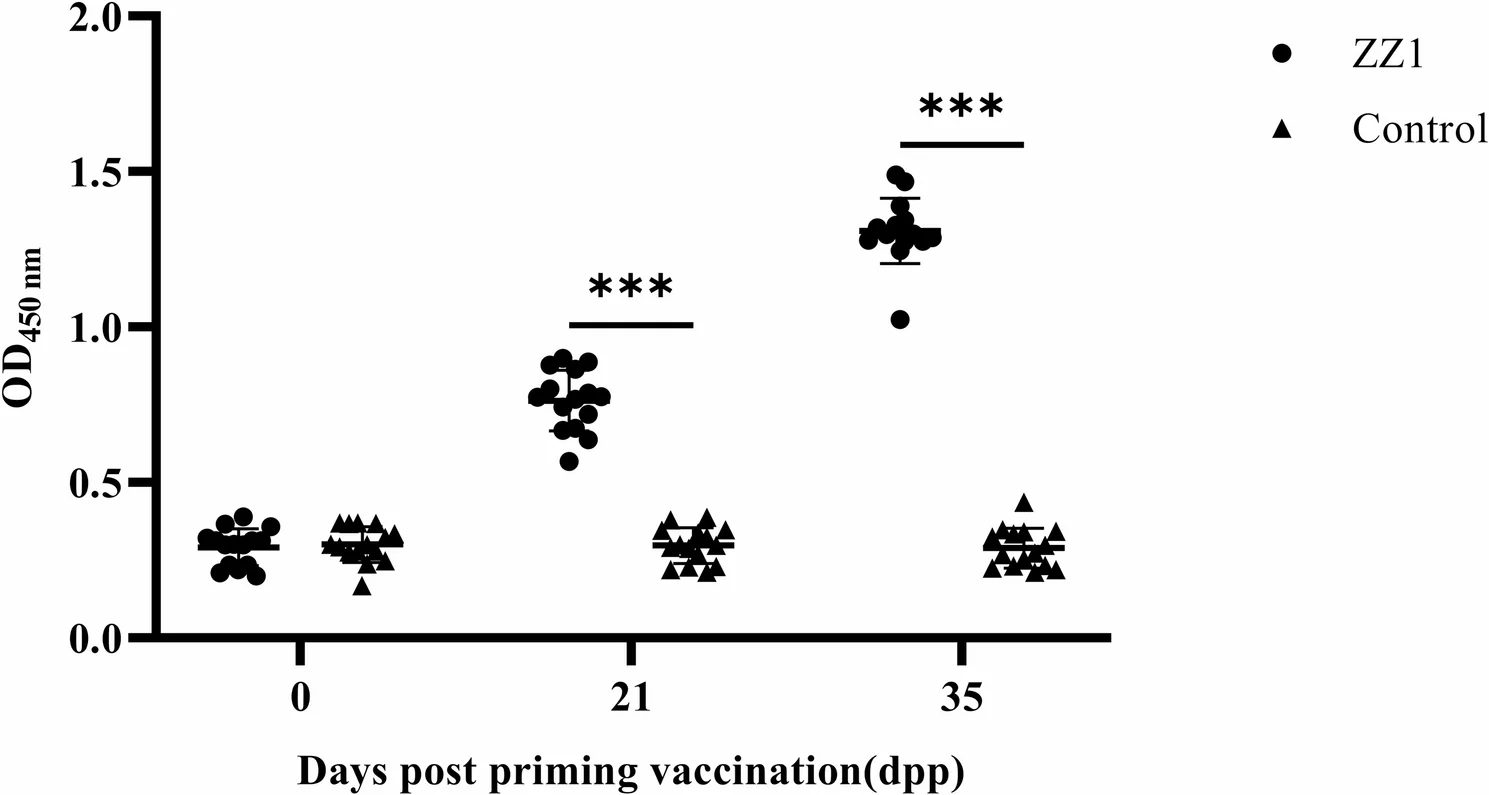
Dynamics of ZZ1-specific serum IgG antibody responses in piglets post-vaccination. Serum collected at day 0 (pre‑vaccination), day 21 (pre‑booster), and day 35 (pre‑challenge). Serum samples were diluted 1:200 for the indirect ELISA. IgG levels measured by indirect ELISA (OD_450_ nm). The solid circles (●) represent the ZZ1-vaccinated group, and the solid triangles (▲) denote the control group (PBS). Data are mean ± SD. Two‑way repeated‑measures ANOVA with Sidak’s post‑hoc test; *** *p* < 0.001 for ZZ1 vs. control at days 21 and 35

To evaluate the in vivo immunogenicity and dynamics of the humoral immune response post-vaccination, ZZ1-specific serum IgG levels were quantified using an indirect ELISA. The results demonstrated that the ZZ1 vaccine elicited a robust and measurable IgG response in piglets by 21 days post-primary immunization, with antibody titers peaking 14 days following the booster dose. Notably, specific IgG levels in the vaccinated group were significantly elevated compared to those in the control group on both days 21 and 35 post-immunization (*p* < 0.001) (Fig. 7). Furthermore, the substantial increase in antibody titers from day 21 to day 35 indicates that the booster immunization effectively amplified the antigen-specific humoral response.

### Challenge protection in vaccinated piglets

Throughout the vaccination period, the immunized piglets exhibited no adverse clinical symptoms, nor were any local reactogenicities (e.g., redness, swelling, or lesions) observed at the injection sites, thereby confirming the excellent in vivo safety and tolerability of the ZZ1 formulation.

**Fig. 8:**
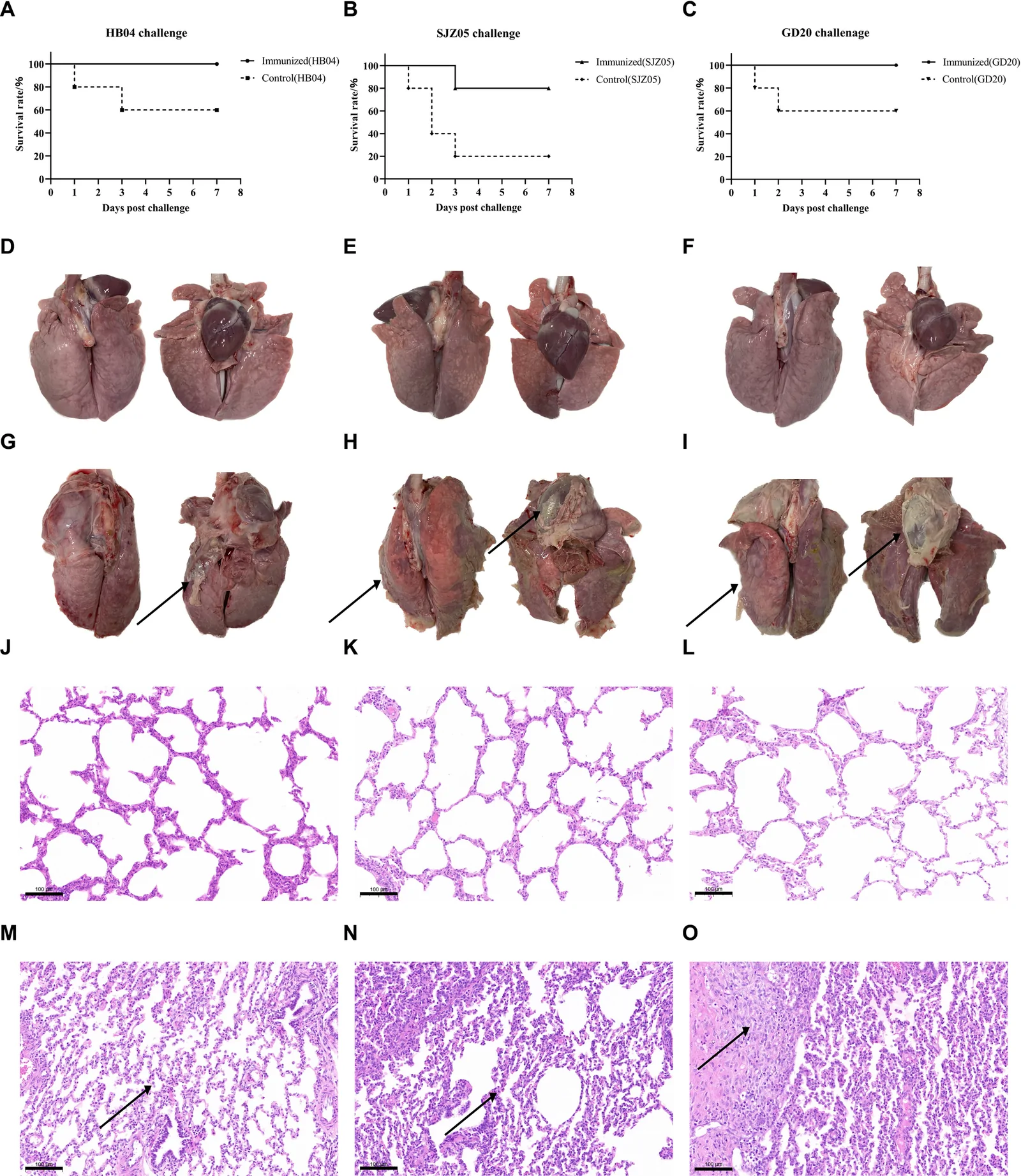
Comprehensive evaluation of ZZ1 vaccine protective efficacy against heterologous*G. parasuis*lethal challenge in a piglet model. **A**–**C** Kaplan‑Meier survival curves post‑challenge: HB04 (serotype 4; 100% vs. 60% survival), SJZ05 (serotype 5; 80% vs. 20%), and GD20 (serotype 13; 100% vs. 60%). **D**–**F** Gross pathology of ZZ1‑vaccinated groups: no lesions. **G**–**I** Control groups: severe fibrinous polyserositis. **J**–**L** Histopathology (H&E) of vaccinated lungs: intact alveolar architecture. **M**–**O** Control lungs: severe disorganization, alveolar atrophy, inflammatory infiltration, and interstitial thickening. Scale bars = 100 μm; arrows indicate lesions

Following virulent challenge, piglets in the unvaccinated control groups (Groups D–F) rapidly developed severe clinical manifestations characteristic of *Glässer’s* disease, including respiratory distress, loss of appetite, paddling movements, lameness, and hind limb paralysis (Table 6). Mortality in the control groups was severe: in Group D, mortalities occurred on days 1 (*n* = 1) and 3 (*n* = 1) post-challenge; in Group E, pigs succumbed on days 1 (*n* = 1), 2 (*n* = 2), and 3 (*n* = 1); and in Group F, one death occurred on each of days 1 and 2 (Fig. 8A–C). Gross pathological examination of these control animals revealed pathognomonic lesions, including the accumulation of yellowish fluid in the thoracic and abdominal cavities, severe pleuropulmonary adhesions with abundant fibrinous exudation, and fibrinous polyserositis involving the joint spaces (Fig. 8G–I). Histopathological evaluation of the lungs further demonstrated severe structural disorganization, alveolar atrophy, massive inflammatory cell infiltration, and marked thickening of the alveolar interstitium (Fig. 8M–O).

**Table 6:** Experimental results of piglets challenged with *G. parasuis*

Groups	Vaccine	Morbidity (%) ^a^	Mortality (%)	Protective Efficacy (%) ^b^
A	ZZ1-ISA201vg	0(0/5)	0(0/5)	100(5/5)
B	ZZ1-ISA201vg	20(1/5)	20(1/5)	80(4/5)
C	ZZ1-ISA201vg	20(1/5)	0(0/5)	80(4/5)
D	PBS-ISA201vg	100(5/5)	40(2/5)	0(0/5)
E	PBS-ISA201vg	100(5/5)	80(4/5)	0(0/5)
F	PBS-ISA201vg	100(5/5)	40(2/5)	0(0/5)

^a^ Morbidity was defined as death or characteristic gross lesions^c^ accompanied by either fever (≥ 40.5 °C for ≥ 2 days) or typical clinical signs^d^

^b^ Protective efficacy was calculated based on morbidity: piglets were considered protected if they exhibited no signs of disease during the observation period post-challenge, or failed to meet the morbidity^a^ criteria

^c^ Characteristic gross lesions: fibrinous pleuritis/pericarditis/peritonitis (fibrinous exudate or adhesions) or arthritis (joint effusion/fibrinous exudate)

^d^ Characteristic clinical signs: ≥1 of depression, coughing, dyspnea, emaciation, lameness, or rough hair coat, lasting ≥ 3 days

Conversely, within the vaccinated test groups, only one mortality was recorded (Group B, day 3), and one piglet in Group C met the morbidity criteria, presenting with characteristic gross lesions accompanied by clinical signs; all other immunized piglets remained clinically healthy and asymptomatic throughout the entire observation period (Fig. 8A–C). Subsequent necropsies of the remaining immunized animals revealed no gross morphological abnormalities in the thoracic/abdominal cavities, joint spaces, or lungs (Fig. 8D–F), a finding further corroborated by the absence of significant histopathological lesions (Fig. 8J–L). Quantitatively, these survival and clinical data demonstrate that the ZZ1 vaccine confers 100% protective efficacy against the *G. parasuis* HB04 isolate (serotype 4), and 80% protective efficacy against both the SJZ05 (serotype 5) and GD20 (serotype 13) isolates. In conclusion, the recombinant ZZ1 vaccine provided robust, highly effective cross-protection against *G. parasuis* serotypes 4, 5, and 13.

## Discussion

*G. parasuis* is an opportunistic pathogen that colonizes the porcine upper respiratory tract and causes *Glässer’s* disease, resulting in substantial economic losses to the global swine industry [1]. The pervasive misuse of antibiotics has accelerated the emergence of antimicrobial resistance, rendering traditional therapeutic interventions increasingly ineffective [35]. MEVs can theoretically overcome the narrow serotype-restricted immunity of traditional bacterins while circumventing biosafety risks associated with live vaccines [7]. To address current limitations in *G. parasuis* prevention, we engineered a MEV through an integrative proteomics and immunoinformatics framework, demonstrating highly promising cross-protective efficacy against serotypes 4, 5, and 13.

Antigen screening fundamentally dictates MEV immunogenicity. Previously, Pang et al. used a pan-genomic approach to identify the *G. parasuis* core genome and construct an epitope vaccine [36]. While pan-genomics can systematically elucidate conserved targets, it carries a theoretical risk of incorporating non-protective antigens, which may affect the focused quality of the immune response [36, 37]. Acknowledging the opportunistic nature of *G. parasuis*, we implemented a differential empirical screening strategy that strictly prioritized OMPs from highly virulent, highly immunogenic strains across multiple serotypes. Through 4D label-free proteomic analysis, we identified 22 conserved OMPs, then rigorously down-selected to eight candidates based on antigenicity and physicochemical stability [38]. This proteome-driven strategy specifically targets in vivo-expressed virulence factors, mitigating the risk of immune tolerance to non-pathogenic proteins and enhancing vaccine specificity. Notably, although our screening framework diverged from that of Pang et al., the convergent identification of peptidoglycan-associated lipoprotein (PAL) in both studies strongly corroborates its pivotal role as a virulence factor and premier vaccine target [39].

OMPs are indispensable for bacterial structural integrity and membrane transport [40]. Due to surface exposure and conservation, they are prime subunit vaccine targets. From the eight selected OMPs, we systematically predicted 6 HTL, 7 CTL, and 11 linear B-cell epitopes. These were concatenated using GPGPG (HTL), AAY (CTL), and KK (B-cell) linkers to prevent junctional neo-epitopes and optimize spatial presentation [25, 41]. The N-terminal TLR2 agonist PSMα4 was fused via a rigid EAAAK linker [26]. The final ZZ1 construct (460 aa) was predicted non-allergenic and non-toxic, with a high antigenicity score of 1.018. Tertiary structure validation confirmed high structural quality (94.10% of residues in favored Ramachandran regions, Z‑score − 8.02) [17]. While the artificial fusion of diverse epitopes poses a theoretical risk of protein misfolding or epitope masking, the robust in vivo antibody responses observed in our animal trials demonstrate that the flexible linker strategy successfully preserved epitope bioavailability. Interestingly, although the IEDB server predicted five conformational B-cell epitopes within ZZ1 [33], the chimeric architecture of an MEV inherently differs from native continuous polypeptides. This suggests that traditional predictive algorithms, predominantly trained on native full‑length proteins, may underestimate the functional presentation of linear epitopes in artificial synthetic constructs—highlighting a need for further experimental data‑driven optimization of these bioinformatic models.

To empirically validate the construct, recombinant ZZ1 (52.3 kDa) was expressed and emulsified with ISA 201 VG adjuvant. Serological analysis confirmed that ZZ1 elicited high titers of antigen‑specific IgG. Most importantly, challenge trials demonstrated that the ZZ1 vaccine conferred 100% protection against the highly virulent HB04 isolate (serotype 4) and 80% protection against SJZ05 (serotype 5) and GD20 (serotype 13). Currently, literature on *G. parasuis* MEVs remains limited. While Pang et al. demonstrated broad in vitro cross‑reactivity using murine antisera, their study lacked virulent challenge assays to confirm definitive protection [36]. Similarly, Dai et al. reported an MEV (Xlc + Ddc) providing 62.5%–87.5% protection in mice [42]. However, *G. parasuis* exhibits strict host specificity for swine. Qi et al. previously highlighted the severe limitations of murine models in replicating *G. parasuis* pathogenesis—a discrepancy we also encountered during preliminary screening [43]. By using the natural piglet host throughout this study, we ensured high clinical relevance and translational reliability. Unlike single‑antigen subunit vaccines that often require multivalent combinations to achieve only partial protection [44], our single recombinant MEV independently achieved 80–100% cross‑serotype protection, simplifying purification and reducing manufacturing costs.

Beyond *G. parasuis*, the integrated proteomics-immunoinformatics pipeline established here—combining comparative proteomics of virulent strains with immunoinformatics-driven epitope selection—offers a generalizable framework for developing MEV against other veterinary bacterial pathogens, especially those facing serotype diversity and poor cross-protection from conventional bacterins.

Despite these promising results, several limitations warrant further investigation. First, while ZZ1 incorporated both HTL and CTL epitopes to engage cellular immunity, our validation primarily quantified humoral responses; future studies should incorporate flow cytometric analyses and cytokine profiling (e.g., IFN-γ, IL-4, IL-17) to characterize cellular immune pathways and memory T-cell proliferation. Second, the current challenge trials were evaluated over an acute observation period; longitudinal studies are required to assess the durability of immunological memory. Finally, although ZZ1 provided robust protection against serotypes 4, 5, and 13, subsequent challenge models should be expanded to include other highly virulent serotypes (such as 1, 10, 12, and 14) to comprehensively define its broad-spectrum potential before large-scale clinical application.

## Conclusions

In summary, this study successfully integrated comparative proteomics and advanced immunoinformatics to rationally design a novel MEV, designated ZZ1, against *G. parasuis*. Through rigorous systematic screening, eight highly conserved OMPs characterized by optimal antigenicity, hydrophilicity, and structural stability were identified. From these core targets, 6 HTL, 7 CTL, and 11 B-cell immunodominant epitopes were systematically extracted and concatenated. Comprehensive in silico validations confirmed that the ZZ1 construct possesses superior antigenicity, robust physicochemical stability, and strict non-allergenic and non-toxic profiles. Crucially, in vivo validation in a natural piglet host model demonstrated that recombinant ZZ1 elicited a potent antigen-specific humoral immune response (IgG) and conferred profound cross-protective efficacy—conferring 100% protection against serotype 4 and 80% against serotypes 5 and 13—against lethal challenge with highly virulent *G. parasuis* strains. Consequently, ZZ1 emerges as a highly efficacious, broad-spectrum prophylactic candidate capable of mitigating *Glässer’s* disease. Furthermore, this work establishes a robust translational framework for epitope-driven subunit vaccine design, providing critical methodological insights to accelerate the development of next-generation veterinary therapeutics.

## Supplementary Information

Additional file 1.

Additional file 2.

Additional file 3.

## Data Availability

All data supporting the findings of this study are included in this paper and its additional files.

## Abbreviations

G. parasuis: Glaesserella parasuis
OMPs: Outer membrane proteins
MEV: Multi-epitope vaccine
HTL: Helper T lymphocytes
CTL: Cytotoxic T lymphocytes
E. coli: Escherichia coli
PSMα4: Phenol-soluble modulator protein α4
TLR2: Toll-like receptor 2
TSA: Tryptic soy agar
TSB: Tryptic soy broth
GRAVY: Grand average of hydropathicity index
PIs: Prominence indices
