# Supplementary material for: Proteomics- and immunoinformatics-based design of a multi-epitope vaccine ZZ1 against Glaesserella parasuis
Source: BMC Vet Res. 2026 Jun 12;22:480. doi: 10.1186/s12917-026-05621-6 (PMC13491728; doi:10.1186/s12917-026-05621-6)

Figure6 B Unlabeled

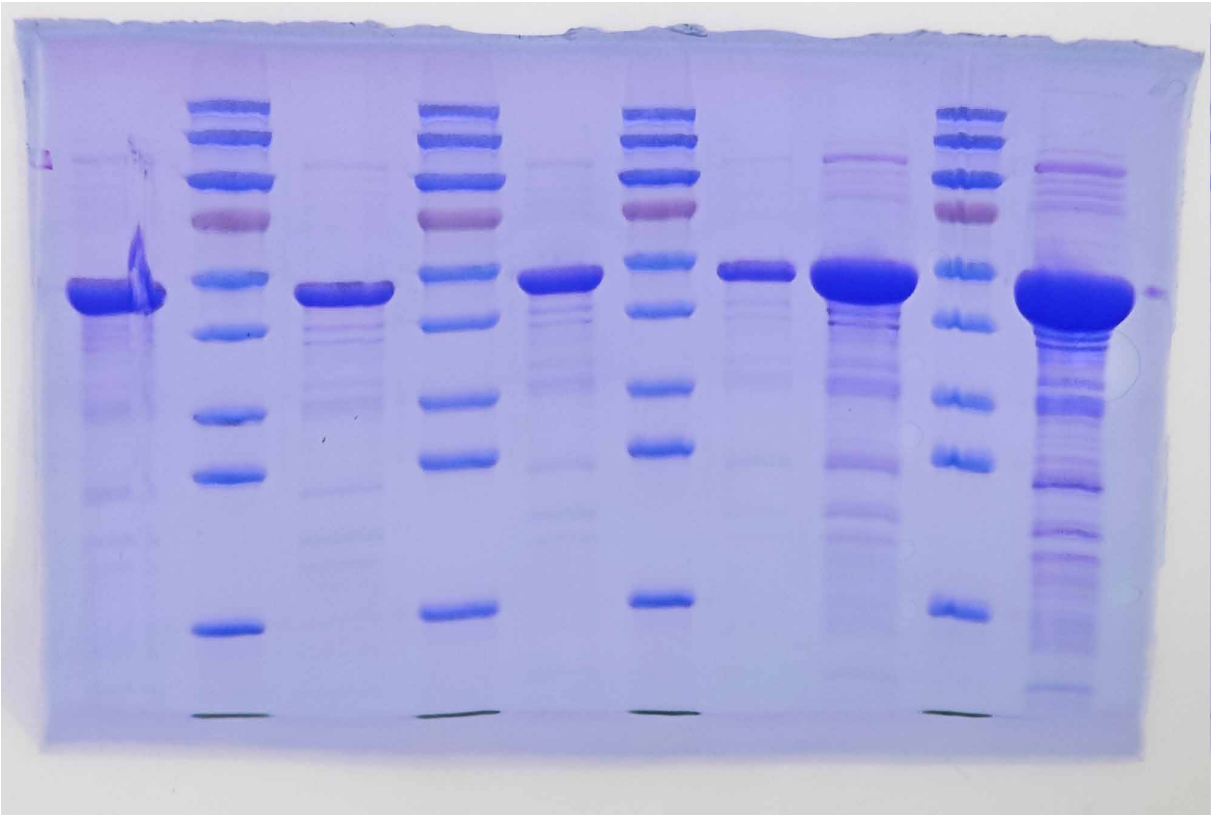

Figure6 B

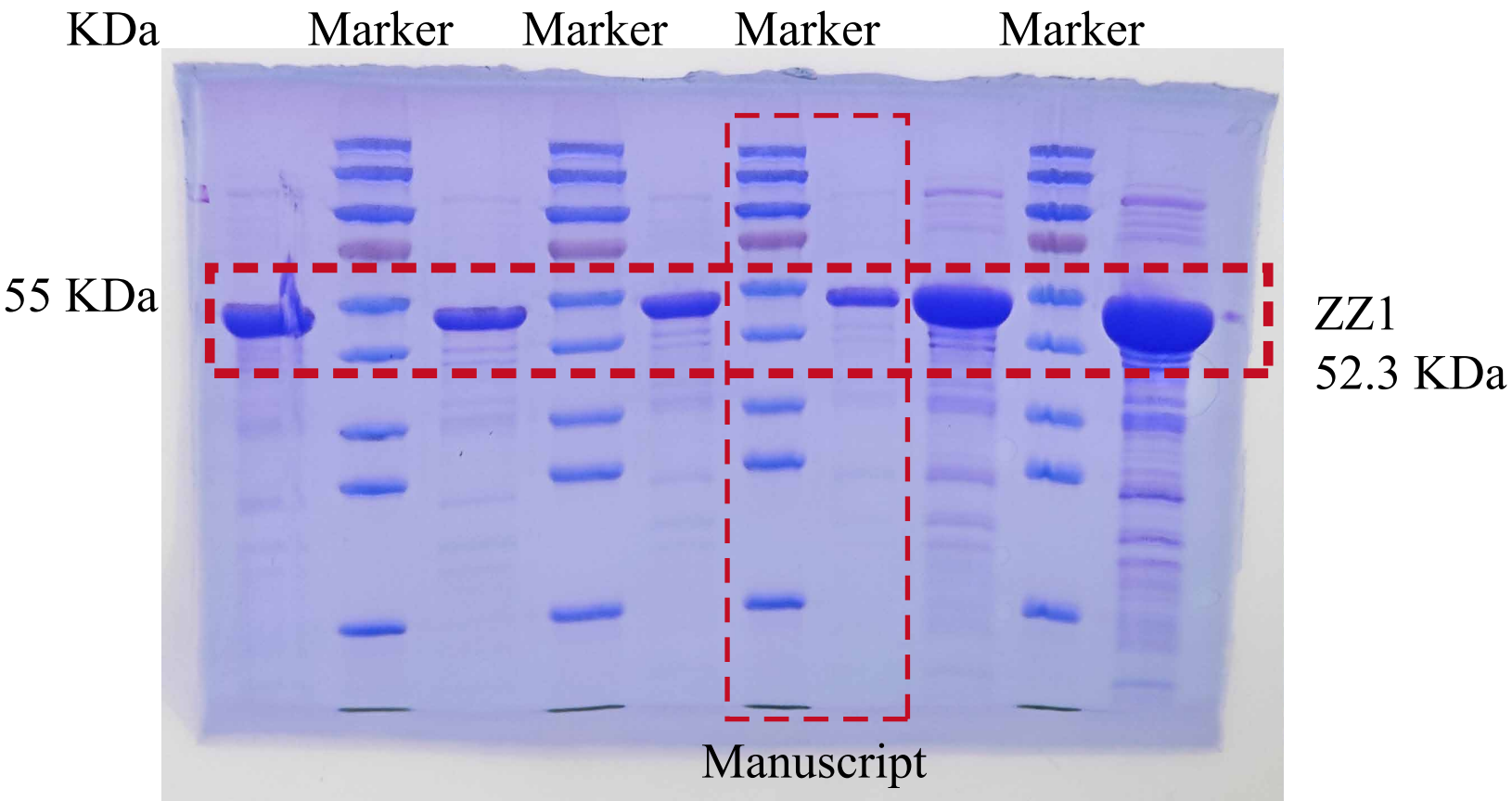

Supplement: Supplementary file 1 [file 12917_2026_5621_MOESM3_ESM.pdf]
