# Supplementary material for: Proteomics- and immunoinformatics-based design of a multi-epitope vaccine ZZ1 against Glaesserella parasuis
Source: BMC Vet Res. 2026 Jun 12;22:480. doi: 10.1186/s12917-026-05621-6 (PMC13491728; doi:10.1186/s12917-026-05621-6)
Supplement: Supplementary file 2 [file 12917_2026_5621_MOESM2_ESM.docx]

**Table S1.** Antigenicity and key physicochemical properties of OMPS.

| NO. | Uniprot ID | Gene | Antigenicity | Theoretical pI | Instability index | Aliphatic index | GRAVY |
| --- | --- | --- | --- | --- | --- | --- | --- |
| A | A0A806JA38 | Pal | 0.7279 | 6.28 | 34.12 | 77.32 | -0.305 |
| B | A0A8A2WC79 | LppB | 0.6218 | 8.86 | 39.96 | 69.85 | -0.405 |
| C | A0A836MCH5 | BamA | 0.4866 | 7.04 | 28.16 | 81.13 | -0.405 |
| D | B8F3L9 | NlpE | 0.2390 | 7.61 | 18.20 | 69.26 | -0.579 |
| E | U4SI14 | BamD | 0.3863 | 5.59 | 29.83 | 78.80 | -0.518 |
| F | B8F4P5 | HAPS_0648 | 0.5791 | 5.65 | 36.21 | 75.90 | -0.545 |
| G | B8F5H6 | RlpB | 0.3502 | 9.14 | 54.53 | 94.63 | -0.235 |
| H | A0A859IIZ4 | FLK62_11795 | 0.6860 | 5.68 | 46 | 69.76 | -0.343 |
| I | A0A859IFM7 | FLK62_04400 | 0.7496 | 5.45 | 36.70 | 70.25 | -0.891 |
| J | U4SC02 | A2U20_02695 | 0.3708 | 8.22 | 40.19 | 74.46 | -0.666 |
| K | A0A859IK31 | FLK62_12140 | 0.5672 | 6.11 | 31.43 | 79.64 | -0.468 |
| L | B8F6K9 | EspP2 | 0.6739 | 6.28 | 25.29 | 74.23 | -0.508 |
| M | T1RQ37 | Wza15 | 0.4669 | 7.73 | 26.86 | 100.49 | -0.109 |
| N | B8F8P8 | VacJ | 0.4913 | 5.11 | 37.31 | 86.09 | -0.283 |
| O | B8F4Z5 | AidA | 0.7066 | 5.04 | 29.05 | 75.07 | -0.495 |
| P | D9IR01 | OmpP5 | 0.6758 | 9.26 | 21.34 | 83.51 | -0.210 |
| Q | A0A1T0AED2 | B0183_00335 | 0.7639 | 8.29 | 31.15 | 101.74 | -0.215 |
| R | F2GDE3 | VtaA7 | 0.9757 | 9.04 | 16.10 | 69.50 | -0.426 |
| S | A0A377IL18 | NCTC4557_01537 | 0.9010 | 9.01 | 12.89 | 57.14 | -0.621 |
| T | A0A0M3U1B8 | TbpB | 0.7893 | 6.31 | 25.18 | 61.65 | -0.633 |
| U | B8F352 | Wza | 0.4511 | 7.73 | 26.14 | 100.76 | -0.092 |
| V | A0A8A2WC79 | J1G50_09015  (LppA) | 0.7448 | 8.51 | 39.62 | 69.09 | -0.894 |
